# Molecular Classification of Soft Tissue and Bone Tumors

**DOI:** 10.3390/diagnostics11122326

**Published:** 2021-12-10

**Authors:** David Creytens

**Affiliations:** 1 Department of Pathology, Ghent University Hospital, Ghent University, 9000 Ghent, Belgium; david.creytens@uzgent.be; Tel.: +32-9-332-3666; 2 Cancer Research Institute Ghent (CRIG), Ghent University, 9000 Ghent, Belgium

Soft tissue and bone tumors constitute a large and heterogeneous group of tumors comprising >100 distinct histological types and subtypes, which are diagnosed and classified using criteria from the World Health Organization (WHO) Classification of Tumors [1]. The diagnosis of soft tissue and bone tumors can be very challenging, due to the large number of separate entities, histologic diversity and rarity of most of these tumor types. The classification of soft tissue and bone tumors has evolved considerably in the past three decades, largely due to major advances in understanding the pathogenetic basis of many of these rare tumors. The identification of characteristic molecular alterations for many of these tumor types (e.g., characteristic gene mutations, gene fusions, and copy number variations) has led to more reproducible and uniform diagnostic criteria, as well as the development of useful ancillary diagnostic tests in soft tissue and bone tumor diagnostic pathology, including immunohistochemistry and molecular diagnostics. 

Some examples of diagnostically important molecular events in soft tissue and bone tumors include *MDM2* gene amplifications, *RB1* gene deletions, *EWSR1* gene rearrangements, *KIT,* and *PDGFRA* gene mutations. 

The sarcomas in which *MDM2* amplification is a hallmark are atypical lipomatous tumor/well-differentiated liposarcoma, dedifferentiated liposarcoma, intimal sarcoma, and low-grade osteosarcoma. In his literature review, Dr. Sciot summarizes the typical clinical, histopathological, immunohistochemical and genetic features of these “*MDM2* amplified sarcomas” [2].

The deletion of the *RB1* gene, a well-known tumor suppressor gene, has been implicated in the tumorigenesis of a particular group of soft tissue neoplasms, including spindle cell/pleomorphic lipoma, atypical spindle cell/pleomorphic lipomatous tumor, pleomorphic liposarcoma, myofibroblastoma, cellular angiofibroma, and acral fibromyxoma. Dr. Libbrecht and colleagues report an updated overview of the currently known morphological, immunohistochemical, and molecular features of this heterogeneous group of “*RB1*-deleted soft tissue tumors” with an emphasis on differential diagnosis [3].

Dr. Flucke and colleagues provide an update on the wide variety of soft tissue and bone entities harboring *EWSR1* gene rearrangements. In their review, the authors present an extensive overview of the clinicopathologic, immunohistochemical, and molecular features and discuss the differential diagnosis of this large, heterogeneous and diagnostically challenging group of “*EWSR1*-rearranged mesenchymal tumors” [4].

Gastrointestinal stromal tumors (GISTs) are the most common mesenchymal tumors of the gastrointestinal tract. The majority of GISTs harbor mutually exclusive *KIT* or *PDGFRA* gain-of-function mutations. Up to 85% of pediatric GISTs and 10–15% of adult GISTs are devoid of these *KIT*/*PDGFRA* mutations and are referred to as “wild-type” GIST. In their update on the molecular genetics of GISTs, Dr. Brcic and colleagues focus on GIST sub-classification based on clinicopathologic and molecular findings and discuss the known and yet emerging prognostic and predictive genetic alterations in this interesting group of soft tissue tumors [5]. 

An increasing number of the more recently described soft tissue tumor entities are defined, for a significant part, based on their specific driving genetic mechanism, supporting their separate classification. An illustration of the latter is the introduction of the “*NTRK*-rearranged spindle cell neoplasms” as an emerging separate entity in the 5th edition of the WHO Classification of Soft Tissue and Bone Tumors [1]. Dr. Siozopoulou and colleagues focus in their review on the diagnostic challenges and the clinical importance of *NTRK* fusions in tumors, with special emphasis on sarcomas [6].

The role of diagnostic immunohistochemistry of soft tissue and bone tumors has also expanded in recent years, with the development of numerous biomarkers based on underlying molecular events. Such biomarkers allow the pathologist to infer the presence of these molecular events and can therefore substitute for other molecular genetic techniques (e.g., fluorescence in situ hybridization, polymerase chain reaction, and next-generation sequencing). In their review, Drs. Anderson and Jo discuss a range of immunohistochemical biomarkers that correlate with molecular alterations in soft tissue and bone tumors, highlighting the accuracy, staining characteristics, and interpretation pitfalls of each antibody [7].

In their review “translating molecular profiling of soft tissue sarcomas into daily clinical practice”, Drs. Jacobs and Lapeire describe, from an oncologist’s point of view, how in the past few years, advanced molecular profiling in soft tissue sarcomas was able to identify specific and often pathognomonic aberrations, deferring standard sarcoma treatment in favor of more targeted treatment [8].

Scattered pieces of evidence suggest the involvement of the PD-1/PD-L1 immune checkpoint in the pathogenesis of osteosarcoma. In their study, Dr. Hashimoto and colleagues used osteosarcoma specimens from cases treated in their hospital to investigate and further characterize the relationship between clinical factors and the expression status of PD-1/PD-L1 immune checkpoint proteins, including CD4 and CD8 [9].

Finally, two reviews of Drs. Choi and Ro and Dr. Akaev and colleagues provide updates on the diagnostic pathology approach to retroperitoneal sarcomas and endometrial stromal uterine tumors and review their key histologic findings and differential diagnoses [10,11].

We hope that readers will appreciate this Special Issue concerning “Molecular Classification of Soft Tissue and Bone Tumors”, elucidating further the diagnostic and clinical importance of molecular pathology in the classification of soft tissue and bone tumors, which remains crucially important for patient management, prognostication, and research efforts.
